# Simoa‐based quantification of DYRK1A in plasma and brain tissue: implications for Alzheimer's disease and Down syndrome biomarker discovery

**DOI:** 10.1002/alz70856_105452

**Published:** 2026-01-07

**Authors:** Idil Yuksekel, Daniella Balduino Victorino, Eugenio Barone, Charlotte Jacob, Debby Van Dam, Jean Maurice Delabar, Nicolas Villain, Kaj Blennow, Marie‐Claude Potier

**Affiliations:** ^1^ Sorbonne Université, INSERM U1127, CNRS 7225, Paris Brain Institute ‐ PBI, Paris, France; ^2^ Sapienza University of Rome, Rome, Rome, Italy; ^3^ University of Antwerpen, Antwerpen, Antwerpen, Belgium; ^4^ Reference Center for Biological Markers of Dementia (BIODEM), Laboratory of Neurochemistry and Behavior, Institute Born‐Bunge, University of Antwerp, Antwerp, Belgium; ^5^ University of GrÖningen, University Medical Center Groningen (UMCG), Department of Neurology and Alzheimer Research Center, GrÖningen, Netherlands; ^6^ Département de Neurologie, Groupe Hospitalier Pitié‐Salpêtrière, AP‐HP Sorbonne Université, Institute of Memory and Alzheimer's Disease, Paris, France; ^7^ Sorbonne Université, INSERM U1127, CNRS 7225, Institut du Cerveau ‐ ICM, Maladie d’Alzheimer, Maladies à Prions, Paris, France, Paris, France; ^8^ Institute of Neuroscience and Physiology, Sahlgrenska Academy, University of Gothenburg, Gothenburg, Sweden; ^9^ Paris Brain Institute, ICM, Pitié‐Salpêtrière Hospital, Sorbonne Université, Paris, France; ^10^ Sahlgrenska University Hospital, Clinical Neurochemistry Laboratory, Mölndal, Sweden

## Abstract

**Background:**

Dual Specificity Tyrosine Phosphorylation‐Regulated Kinase 1A (DYRK1A) has been implicated in Alzheimer's disease (AD) pathology. Using Meso Scale Discovery (MSD) technology, we previously demonstrated that individuals with AD, Down syndrome with AD (DS‐AD), or tauopathies exhibit reduced plasma DYRK1A levels compared to controls (Delabar et al., 2023). To further evaluate DYRK1A as a potential biomarker, we developed and optimized a homebrew DYRK1A immunoassay using the ultrasensitive Single Molecule Array (Simoa HD‐X platform). This assay was applied to measure DYRK1A levels in plasma and brain homogenates from AD mouse models and plasma samples from individuals with DS and DS‐AD.

**Method:**

To assess the impact of DYRK1A overexpression in the context of AD‐related biomarkers, we analyzed DYRK1A levels using Simoa in wild‐type controls and AD mouse models (APP23 or P301S), with or without DYRK1A overexpression. Additionally, EDTA plasma from healthy controls and age‐matched individuals with DS, with or without AD, was analyzed using the developed immunoassay.

**Result:**

The limit of detection (LOD) and limit of quantification (LOQ) for the assay were 0.021 pg/mL and 0.069 pg/mL, respectively. DYRK1A levels in both plasma and brain homogenates from mouse models exhibited a dose‐dependent change, with significantly higher levels in DYRK1A‐overexpressing mice compared to non‐overexpressing counterparts. The assay was also successfully applied to human cohorts, demonstrating its potential clinical utility. Ongoing experiments aim to quantify longitudinal changes in DYRK1A levels in AD mouse models to further evaluate its biomarker potential.

**Conclusion:**

This study strengthens the evidence for DYRK1A as a potential biomarker for AD and DS‐AD, utilizing ultrasensitive Simoa technology for detection. Future investigations will determine whether longitudinal DYRK1A level changes correlate with AD progression and aging, aiding in early biomarker discovery and potential therapeutic targeting.

